# Comparative quantitative proteomic analysis of disease stratified laser captured microdissected human islets identifies proteins and pathways potentially related to type 1 diabetes

**DOI:** 10.1371/journal.pone.0183908

**Published:** 2017-09-06

**Authors:** Julius O. Nyalwidhe, Wojciech J. Grzesik, Tanya C. Burch, Michele L. Semeraro, Tayab Waseem, Ivan C. Gerling, Raghavendra G. Mirmira, Margaret A. Morris, Jerry L. Nadler

**Affiliations:** 1 Department of Microbiology and Molecular Cell Biology, Eastern Virginia Medical School, Norfolk, Virginia, United States of America; 2 Leroy T. Canoles Jr. Cancer Research Center, Eastern Virginia Medical School, Norfolk, Virginia, United States of America; 3 Department of Internal Medicine, Eastern Virginia Medical School, Norfolk, Virginia, United States of America; 4 Division of Endocrinology, Diabetes and Metabolism, The University of Tennessee Health Science Center, Memphis, Tennessee, United States of America; 5 Department of Pediatrics, Center for Diabetes and Metabolic Diseases, Indiana University, Indianapolis, Indiana, United States of America; 6 The Strelitz Diabetes Center, Eastern Virginia Medical Center, Norfolk, Virginia, United States of America; Baylor College of Medicine, UNITED STATES

## Abstract

Type 1 diabetes (T1D) is a chronic inflammatory disease that is characterized by autoimmune destruction of insulin-producing pancreatic beta cells. The goal of this study was to identify novel protein signatures that distinguish Islets from patients with T1D, patients who are autoantibody positive without symptoms of diabetes, and from individuals with no evidence of disease. High resolution high mass accuracy label free quantitative mass spectrometry analysis was applied to islets isolated by laser capture microdissection from disease stratified human pancreata from the Network for Pancreatic Organ Donors with Diabetes (nPOD), these included donors without diabetes, donors with T1D-associated autoantibodies in the absence of diabetes, and donors with T1D. Thirty-nine proteins were found to be differentially regulated in autoantibody positive cases compared to the no-disease group, with 25 upregulated and 14 downregulated proteins. For the T1D cases, 63 proteins were differentially expressed, with 24 upregulated and 39 downregulated, compared to the no disease controls. We have identified functional annotated enriched gene families and multiple protein-protein interaction clusters of proteins are involved in biological and molecular processes that may have a role in T1D. The proteins that are upregulated in T1D cases include S100A9, S100A8, REG1B, REG3A and C9 amongst others. These proteins have important biological functions, such as inflammation, metabolic regulation, and autoimmunity, all of which are pathways linked to the pathogenesis of T1D. The identified proteins may be involved in T1D development and pathogenesis. Our findings of novel proteins uniquely upregulated in T1D pancreas provides impetus for further investigations focusing on their expression profiles in beta cells/ islets to evaluate their role in the disease pathogenesis. Some of these molecules may be novel therapeutic targets T1D.

## Introduction

Type 1 diabetes (T1D) is a chronic inflammatory disease that is characterized by autoimmune destruction of insulin-producing pancreatic beta cells [1–2]. The triggering event (s) leading to beta cell damage are not known; however, multiple studies have linked a possible viral infection to islet autoimmunity and T1D. Our long term objective is to identify novel protein signatures that distinguish the Islets from patients with T1D from the pancreas from patients who are autoantibody positive without symptoms of diabetes (AAb+), and from patients with no evidence of disease (ND). Laser capture microdissection (LCM) allows for the isolation of enriched cell populations from heterogeneous tissue guided by microscopic visualization. LCM can be used to harvest the cells of interest directly resulting in histologically enriched cell populations [3–6]. In our current study, we have focused on proteomic characterization of laser capture microdissected islets that should be more reflective of the micro-environment of beta cells, as compared to total pancreatic sections. The ultimate objective is the identification of beta cell stress markers and any microorganisms that may have a role in the etiology of T1D.

In this study, we have used LCM for isolation of pancreatic islets from the Network for Pancreatic Organ Donors with Diabetes (nPOD) pancreata. The LCM captured islets from normal and disease stratified were then investigated by high-resolution, accurate-mass (HR/AM) liquid chromatography mass spectrometry (LC/MS/MS) for qualitative and quantitative proteomic analysis to detect differential protein expression in disease stratified pancreas tissue obtained from the nPOD consortium. LCM was performed on pancreas tissue sections from organ donors without diabetes, with T1D-associated autoantibodies in the absence of diabetes, and with T1D. We identified proteins that are significantly differentially regulated in T1D LCM islets compared to the other two groups. These proteins have diverse biological functions, such as inflammation, metabolic regulation, and autoimmunity, all of which are pathways linked to the pathogenesis of T1D. The identified islet proteomes, protein-protein interaction networks, and enriched functional categories presented in the different disease states provide novel insights that should enhance our understanding of the etiology of T1D, and provide impetus for further investigations focusing on their expression profiles in beta cells to evaluate their role in T1D disease. Some of these proteins are likely involved in beta cell stress, as well as T1D development and pathogenesis. These molecules could also be potential biomarkers for T1D and targets for development of novel therapeutics. In addition, this optimized LCM-LC-MS method will be applicable to additional future targeted proteomic analysis of nPOD tissue samples in studies focusing on specific molecules and molecular pathways.

## Materials and methods

### Materials, chemicals and reagents

Laser Capture Microdissection slides and LCM caps were obtained from Thermo Fisher Scientific (Rockford, IL, USA). LCM Liquid Tissue MS Preparation Kit was purchased from Expression Pathology Inc (Rockville, MD, USA). Micro-BCA Protein Assay Kit, dithiothreitol (DTT) and high purity grade solvents including acetonitrile, water and formic acid were from Thermo Fisher Scientific (Rockford, IL, USA). Ammonium bicarbonate and Iodoacetamide were purchased from Sigma-Aldrich (St. Louis, MO, USA). Sequencing Grade Trypsin was purchased from Promega Corporation (Madison, WI, USA).

### Human tissue samples

Pancreatic tissue sections were obtained from optimal cutting temperature (OCT) embedded tissue from deceased organ donors provided by nPOD, University of Florida, Gainesville, Florida, USA, in accordance with ethical regulations [7–8]. These samples do not fall under human subjects research and the study was classified as exempt by the Eastern Virginia Medical School Institutional Review Board (IRB). Tissue samples were obtained from three groups: nondiabetic donors (ND), non-diabetic donors with type 1 diabetes-associated autoantibodies (AAB+) and donors with type 1 diabetes (T1D). Comprehensive qualitative and quantitative proteomic analyses were performed on five ND cases and four cases from AAB+ and T1D cases. The nPOD cases classification criteria are described under S1 Table.

### Laser capture microdissection and protein extraction

For LCM, serial 10 μm-thick OCT embedded frozen sections were prepared from pancreas blocks and attached to either polyethylene naphthalate (PEN) membrane slides or glass-membrane slides that allow for the utilization of both infrared (IR) laser capture and ultraviolet (UV) laser cutting. The tissue samples were processed as follows: Incubated in 70% ethanol for 1 min; rinsed in molecular biology grade water (5 dips); and then incubated sequentially in ethanol at the following concentrations 70%, 95%, and 100% for 1 min each followed by 100% ethanol for 5 min (all steps at 4^o^ C) to dehydrate the samples. The samples were then air-dried for 5–10 min at RT before capture. LCM of islets was performed using an Applied Biosystems ArcturusXT LCM System equipped with dual IR and UV lasers (Thermo Fisher Scientific) according to manufacturer’s instructions. The utilization of the two lasers working together allows for the efficient and more specific isolation of cells and tissue without any detrimental effects on their morphology or integrity of their biological content. The IR laser helps to capture the cells of interest, and the UV laser microdissects the captured cells preventing any significant contamination of captured material with adjacent acinar tissue. For these studies, unstained tissue section islets were identified by their unique and specific intrinsic florescence behavior that is observed upon UV illumination of the specimen that can be detected using triple filter. Typically, the UV cut and IR capture settings were as follows: spot spacing, 110; spot diameter 30–50; spot power 60–70; spot duration 17–20; UV cut speed 900 microns/second. The islets were collected on CapSure LCM Caps (typically, about 1.5–2 mm^2^ of islet area for a single cap) which were then placed onto 0.5 ml PCR tubes and stored at -80°C until use. Protein extraction was performed using the Liquid Tissue MS Protein Prep Kit (Expression Pathology, Rockville, MD) according to the manufacturer's protocol. Briefly, the LCM cap films containing approximately 3 x10^4^ cell equivalents (estimates were based on the thickness and area of the captured islets tissue) were transferred into 20 μl of liquid tissue buffer in a 1.5 ml low protein binding reaction tube and centrifuged at 10,000 x g for 2 minutes to pellet the film. The islet proteins were extracted by heating the mixture at 95°C for 90 min with intermittent mixing at 20 min intervals. After 90 min, the samples were centrifuged at 10,000 x g for 1 minute before cooling in ice for 2 min. The equivalent of 1:50 trypsin was added to the extracted protein and incubated at 37°C for 18 hours to generate tryptic peptides. After the trypsin digestion step, an aliquot of the generated peptides was used in a Micro BCA assay to determine the peptide concentrations. The remaining peptides were reduced (10mM DDT) and alkylated (35mM iodoacetamide) and stored at -80°C prior to use for mass spectrometry analysis.

### Liquid chromatography and mass spectrometry

LC-MS analysis of digested samples was conducted as we have previously described [9]. Tryptic peptides were solubilized in normalized volumes of 0.1% formic acid (FA)/H2O before determining their concentrations using the Micro BCA assay. The concentrations of the samples were adjusted to 0.5 μg/μl in 0.1% formic acid. 2 μg of the tryptic peptides from each of the samples were injected and analyzed on a Q-Exactive Orbitrap mass spectrometer coupled to an Easy NanoLC-1000 system optimized under the following conditions: LC solvents: buffer A, 0.1% formic acid/water; buffer B, 0.1% formic/acetonitrile; column, Thermo Scientific™ EASY-Spray™, 75μm x 150mm C18 column; flow rate 400 nl/min; LC separation, total 120 min stepped linear gradient, 0–2 min 2% B; 2% to 40% B in 112 minutes, followed by 95% B in the next 3 minutes and holding at 95% B for 5 minutes. The MS data was acquired using data-dependent acquisition with following optimized settings: dynamic exclusion (DE = 1); top 12 higher energy collision induced dissociation (HCD); resolving power set at 70,000 for the full MS scan and 17,500 for the MS/MS scan at m/z 200 as we have previously described [9]. Each individual sample was run in randomized triplicate or duplicate LC/ESI-MS/MS analyses. Two blank runs injecting 0.1% formic acid were included before every sample injection to eliminate carry-over between the runs. The acquired high-resolution MS and MS/MS peptide spectra were imported to MaxQuant for further analysis.

### Database searching and label free quantification

High resolution Q-Exactive.RAW MS files were imported to MaxQuant (version 1.5.2.8) [10] with Andromeda as search engine for protein identification/quantitation [11]. LCM samples from the three groups were analyzed as one set and aligned. Analysis of MS spectra was performed using the following parameters: acetylation of the protein N-terminus and oxidation of methionine as variable modifications; carbamidomethylation of cysteine as fixed modification. UniProt-SwissProt human canonical database (version 2016, canonical proteome; 20 198 identifiers) was selected as FASTA file. Seven amino acids were selected as minimum peptide length. Match between runs option was kept as default (match time window: 0.7 min; alignment time window: 4 min). Label free quantitation (LFQ) was enabled and LFQ minimum ratio count was set to 1. Remaining options were kept as default [10, 11].

### Data and statistical analysis

Perseus, which comprises of a comprehensive portfolio of statistical tools for high-dimensional omics data analysis covering normalization, pattern recognition, time-series analysis, cross-omics comparisons and multiple-hypothesis testing of data generated in MaxQuant [12], was used to compare protein expression in the 3 sample groups. “ProteinGroups.txt” was imported from data generated in MaxQuant as described in the preceding section [12]. Peptides were filtered for posterior error probability (< 0.05) and proteins for Q-value (< 0.05). Possible contaminants and reversed sequences were excluded [12]. The detailed protocol for label free quantitation is described in detail by Luber et al. [13]. MaxQuant derived LFQ normalized protein/ peptide intensity were Log2 transformed before further analyses. This label free algorithm takes the maximum number of identified peptides for a specific protein identified between any two samples and compares the intensity of these peptides to determine peptide ratios [13]. Protein abundance was computed using median values of all peptide ratios of certain protein [13]. The peptides that were used for further analysis fulfilled the following criteria: (i) peptides were uniquely assigned to one protein group; (ii) peptides were not identified as in the decoy data base derived from reversed sequences of all peptides in the database. Only peptides with abundance data in at least 50% of the samples were included in the statistical analysis. Proteins without peptide quantitative values in more than half of the triplicate and duplicate measurements from the 13 biological replicates were excluded from the analyses. Data imputation to replace log2 transformed LFQ missing values was done by normal distribution. Using this approach, missing values were imputed using normal distribution to simulate peptide expressions below the detection limit. The approach makes the assumption that in proteomics experiments, low expression proteins give rise to missing values [12]. In this approach, a Gaussian distribution with a median shifted from the measured data distribution results in accurate imputation of such missing values [12]. Two sample t-test was performed on the abundances to confirm differentially expression patterns of these proteins (p<0.05, permutation-based false discovery rates (FDR = 0.05) using Perseus [12, 14]. The combined score probability score was generated as the product of the two tests and the values expressed in a–log10 scale as calculated in Perseus [12]. Volcano plots were used to visualize the results of the t-test. Significantly differentially regulated proteins between AAB+, T1D and control ND islet samples were identified based on the permutation based FDR calculations.

### Protein pathway analysis

Functional classification for the identified proteins was performed using the Panther database (http://www.pantherdb.org) [15]. GO annotation of the identified proteins was done using the NCBI’s DAVID analysis tool (V6.7: http://david.abcc.ncifcrf.gov). GO_CC_FAT, GO_BP_FAT, and GO_MF_FAT algorithms were used [16–17]. The default parameters of the GO enrichment searches were used with a count threshold set at 2 and the EASE (p-value) threshold set at 0.1; fold enrichment as well as FDR values were displayed in search results. Differentially expressed proteins were analyzed using Ingenuity Pathway Analysis (IPA) (Ingenuity Systems: http://www.ingenuity.com). Information stored in the Ingenuity Pathways Knowledge Base was used to identify and predict top canonical pathways, top upstream regulators, top diseases and bio functions and molecular networks.

### Immunofluorescence and confocal microscopy

Formalin fixed paraffin embedded tissue sections from nPOD were processed for immunofluorescence assay as follows. Sections were paraffinized in xylene followed by rehydration in descending concentrations of ethanol in water. Sections were then subject to heat induced epitope retrieval for twenty minutes at 95°C (HIER Buffer, Thermo Scientific). Slides were then blocked with 5% BSA for one hour, followed by primary antibody incubation overnight at 4°C in a low evaporation chamber. The primary antibodies that were used were: S100A9 (ab63818, 1:50), insulin (ab7842, 1:200), and glucagon (ab82270, dilution (1:50). All the antibodies were from Abcam (Cambridge, MA). After three 15 minute washes, sections were stained with the appropriate secondary antibodies conjugated with CY2 or Cy3 at 1:100 dilution for 90 minutes at room temperature in the dark. Slides were washed three times for 15 minutes and mounted with Cytoseal XYL (Thermo Scientific) before imaging using a Zeiss Axiophot epifluorescent microscope.

## Results and discussion

**Fig 1:**
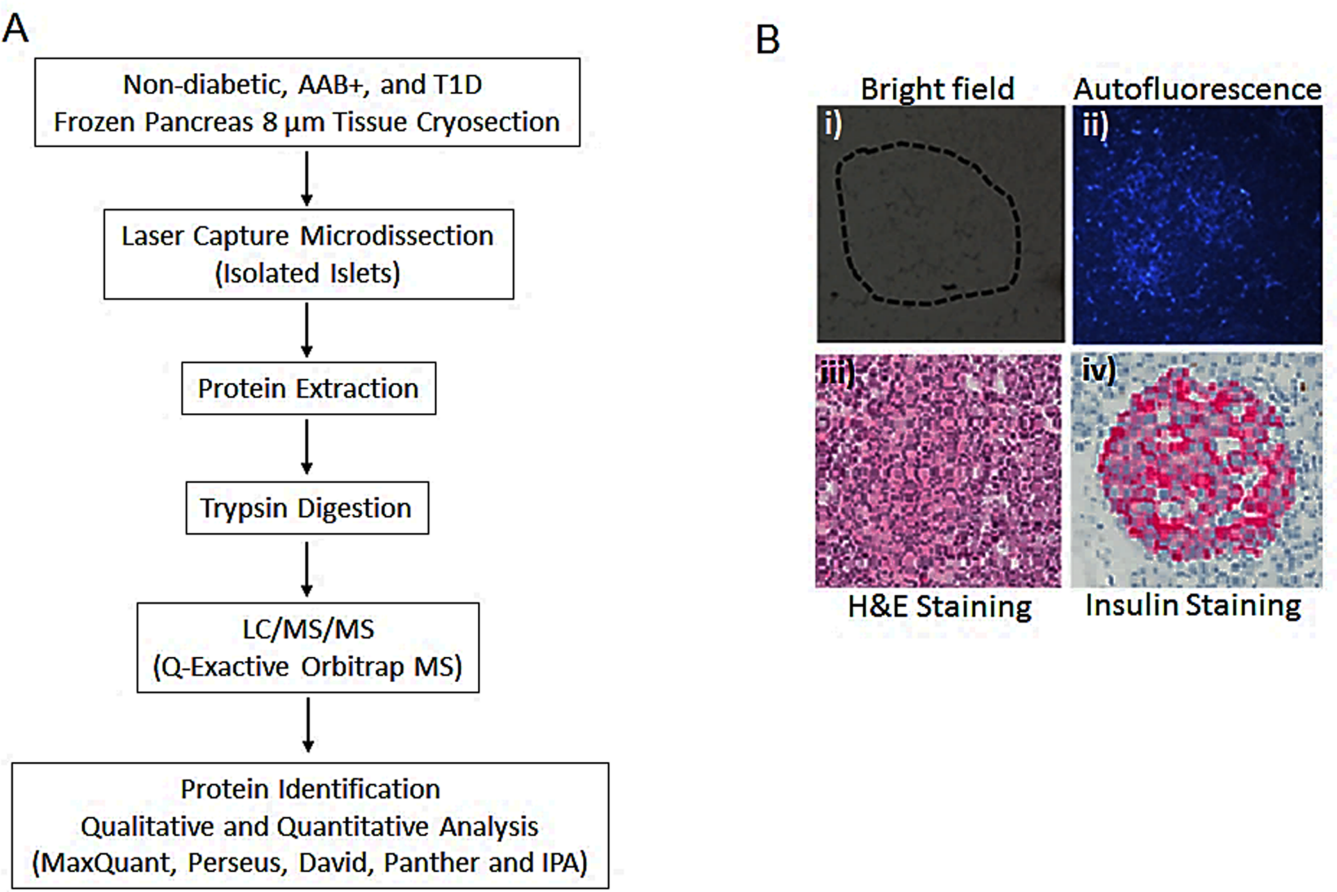
**A). Workflow summarizing the LCM islet proteome characterization. B)**. Tissue sections from nPOD human pancreas examined by microscopy under i) bright field; ii) autofluorescence; iii) stained with hematoxylin and eosin and iv) stained with insulin.

In this study, we report a robust quantitative proteomic analysis approach for the identification of human islet proteins that are associated with type 1 diabetes. This pilot biomarker discovery pipeline is divided into 4 main stages. The first stage consists of laser capture microdissection to harvest highly enriched islet samples from disease stratified human pancreas tissues. The LCM enrichment minimizes interference from the background exocrine pancreas proteome and allows for a more precise quantitation of the islet proteome both in normal and diseased state. The second stage involves the generation of high resolution high mass accuracy raw nLC-MS/MS profiling data; the third stage includes both upstream sample processing steps that include multiple chromatographic peak and spectral alignment, peptide and protein identification, and quantitation and subsequent statistical analysis steps. The fourth and last stage involves GO enrichment and pathway analysis to identify molecular targets and pathways that are associated with T1D disease initiation and progression. A schematic chart summarizing the experimental strategy is shown in Fig 1A.

### LCM capture of islets and morphology and insulin content of islets

All the OCT embedded pancreatic sections from the nPOD cases are stained for pancreatic markers (including insulin and glucagon) as a standard histopathological evaluation. The insulin staining strongly correlated with the auto florescence on unstained sections that is specifically observed in islets using UV illumination. This intrinsic property was used to guide the capture of islets from pancreas tissue samples. It is important to note that the morphology of the pancreas tissues and the isolated islets that were used in this study were very well preserved. Fig 1B shows representative examples of images of pancreas tissue sections observed under bright field, UV illumination, H&E staining and insulin staining.

### Protein expression profiles of T1D, AAB+ and control ND islets

The main objective was to characterize the proteome of islets derived from disease-stratified human pancreata to identify differentially expressed proteins that may play a role in the etiology and pathogenesis of T1D. It is important to note that known endocrine hormones including insulin, glucagon and somatostatin are detected in these analyses. We have identified a total of 2032 different protein groups and 1491 protein groups fulfilled the criteria for comparative quantitative analysis described under the data and statistical analysis in the materials and methods section.

**Fig 2:**
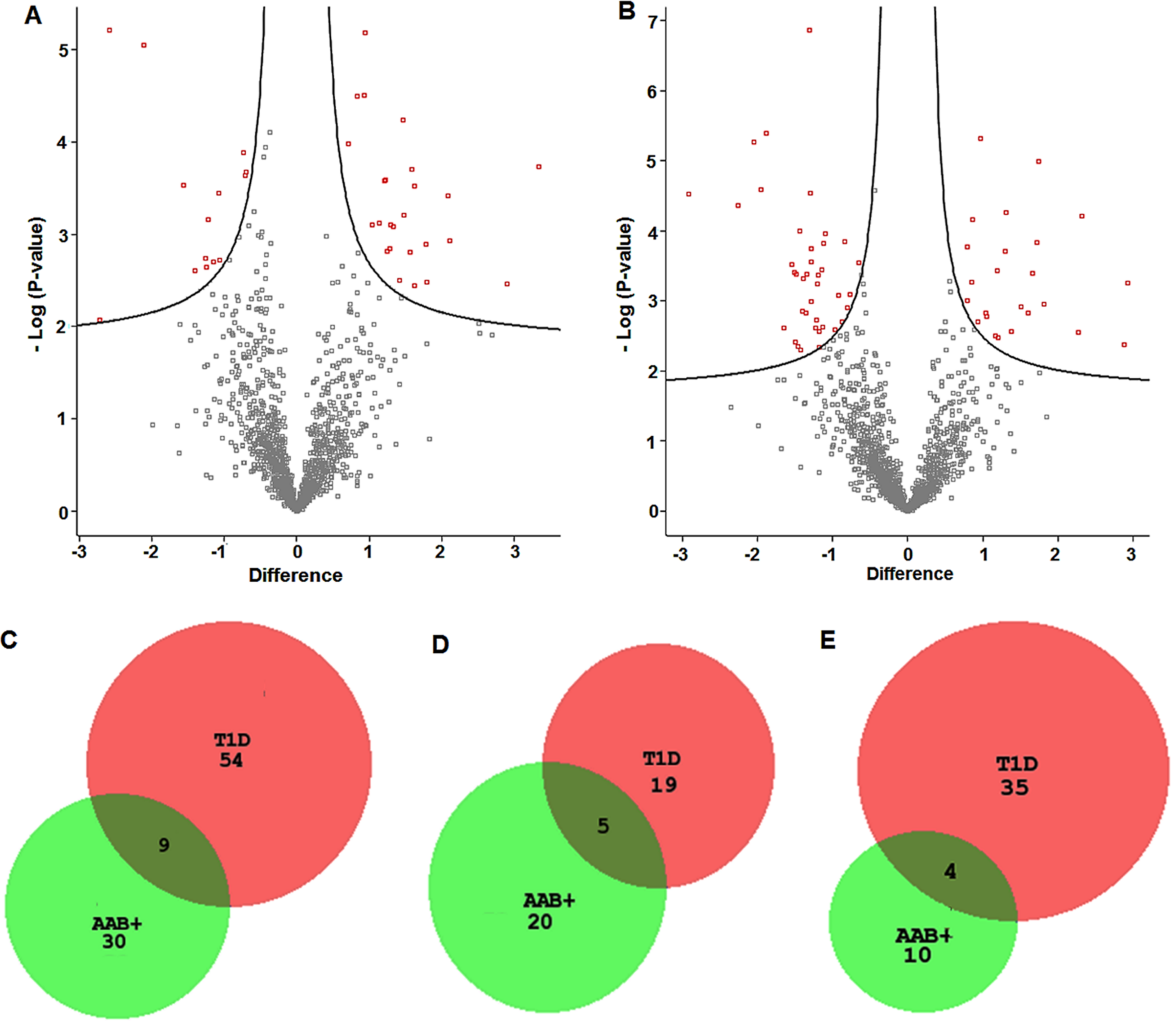
Volcano plots and Venn diagrams showing protein expression in AAB+ and T1D cases versus non-diabetic cases. A). Volcano plot of protein expression in islets from ND versus AAB+. B). Volcano plot of protein expression in islets from ND versus T1D. Volcano plots were constructed using fold-change and p values, enabling visualization of the relationship between fold change and statistical significance. The red boxes in volcano plots represent differentially proteins with statistical significance between ND vs AAB+ and T1D cases. C). Comparison of differentially expressed proteins between AAB+ and T1D versus controls. D) Significantly downregulated proteins between AAB+ and T1D versus controls. E). Significantly upregulated proteins between AAB+ and T1D versus controls.

Perseus was used to analyze differential protein expression between the non-diabetic cases versus autoantibody positive and type 1 diabetes cases. The volcano plots summarizing the expression profiles of proteins in the three sample groups are shown in Fig 2A and 2B. For the AAb+ cases, 39 proteins were differentially regulated compared to the no-disease group, with 25 upregulated and 14 downregulated proteins (S2 Table). For the T1D samples, 63 proteins were differentially expressed, with 24 upregulated and 39 downregulated, compared to the no disease controls (S3 Table). Nine proteins are differentially regulated in both T1D and AAB+, with 5 upregulated and 4 downregulated proteins. A master table that shows the protein/peptide intensities for all the peptides that were quantified in the study is provided (S4 Table). Fig 2C shows the Venn diagram comparison of the differentially expressed proteins between AA+ and T1D islets. Fig 2D shows the comparison of upregulated proteins between AAB+ and T1D islets, and Fig 2E shows the comparison of down regulated proteins between AAB+ and T1D islets.

**Fig 3:**
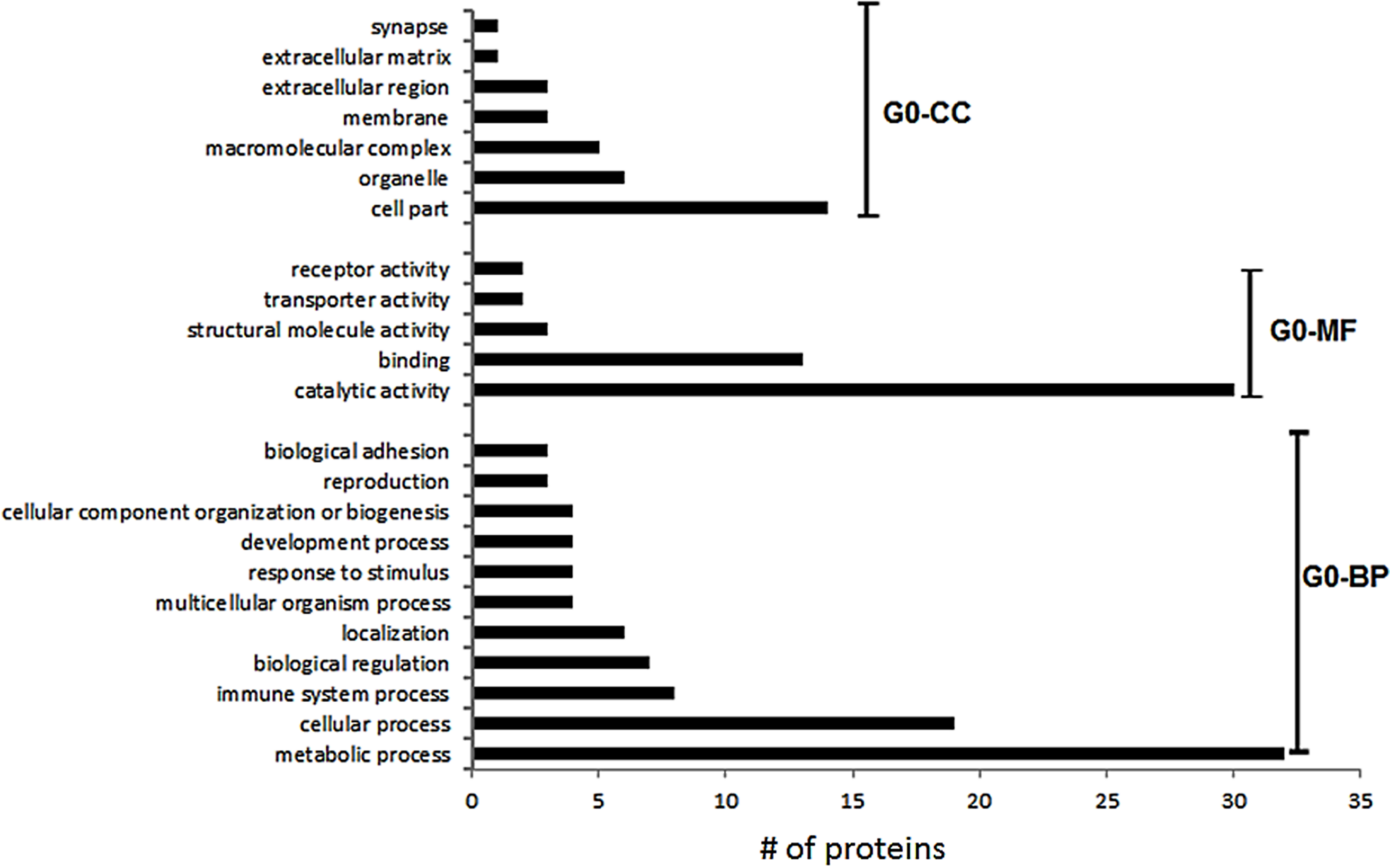
Gene ontology enrichment for differentially proteins between ND versus T1D cases. The GO analysis was performed using Panther [15]. GO-CC denotes Cellular Component, GO-MF denotes Molecular Function and GO-BP, Biological Processes.

The biological processes, molecular functions and cellular components of the 63 identified and quantifiable protein groups were determined using the Panther database [15] and the results are summarized in Fig 3.

The differentially regulated proteins include those that are associated with different metabolic processes, including glycolysis/ gluconeogenesis, oxidative phosphorylation, and secretory granules. Functional annotation enrichment of these differentially expressed proteins was done using David [16–17]. The significance threshold for the enrichment values was set to 2.0. David functional annotation clusters with enrichment scores for differentially regulated proteins in T1D are shown in S5 Table. The significant annotations include: ***Cluster i*** includes GO: 0070062, extracellular exosome; GO: 1903561, extracellular vesicle; GO:0043230, extracellular organelle with an enrichment score of 7.46; ***Cluster ii*** includes GO:0016052, carbohydrate catabolic process with an enrichment score of 4.65; ***Cluster iii*** includes GO:0019752, carboxylic acid metabolic process with an enrichment score of 4.18; ***Cluster iv*** includes GO:0006090, pyruvate metabolic process with an enrichment score of 4.09; ***Cluster v*** includes GO:0046128, purine ribonucleoside metabolic process with an enrichment score of 3.31; ***Cluster vi*** includes GO:0046128, purine ribonucleoside metabolic process with an enrichment score of 3.10; ***Cluster vii*** includes GO:0006096, glycolytic process with an enrichment score of 2.94; ***Cluster viii*** includes GO:0002790, peptide secretion process with an enrichment score of 2.41; ***Cluster ix*** includes GO:0009167, purine ribonucleoside monophosphate metabolic process with an enrichment score of 2.34 and ***Cluster x*** includes GO:0006006, glucose metabolic process with an enrichment score of 2.02. The same analyses were performed using the proteins from AAB+ islets and the results are summarized in S6 Table. There are only two clusters that are beyond the set significance threshold ***Cluster i*** includes GO: 0070062, extracellular exosome; GO: 1903561, extracellular vesicle; GO: 0043230, extracellular organelle with an enrichment score of 6.77; ***Cluster ii*** includes GO: 0016052, carbohydrate catabolic process with an enrichment score of 2.34.

**Fig 4:**
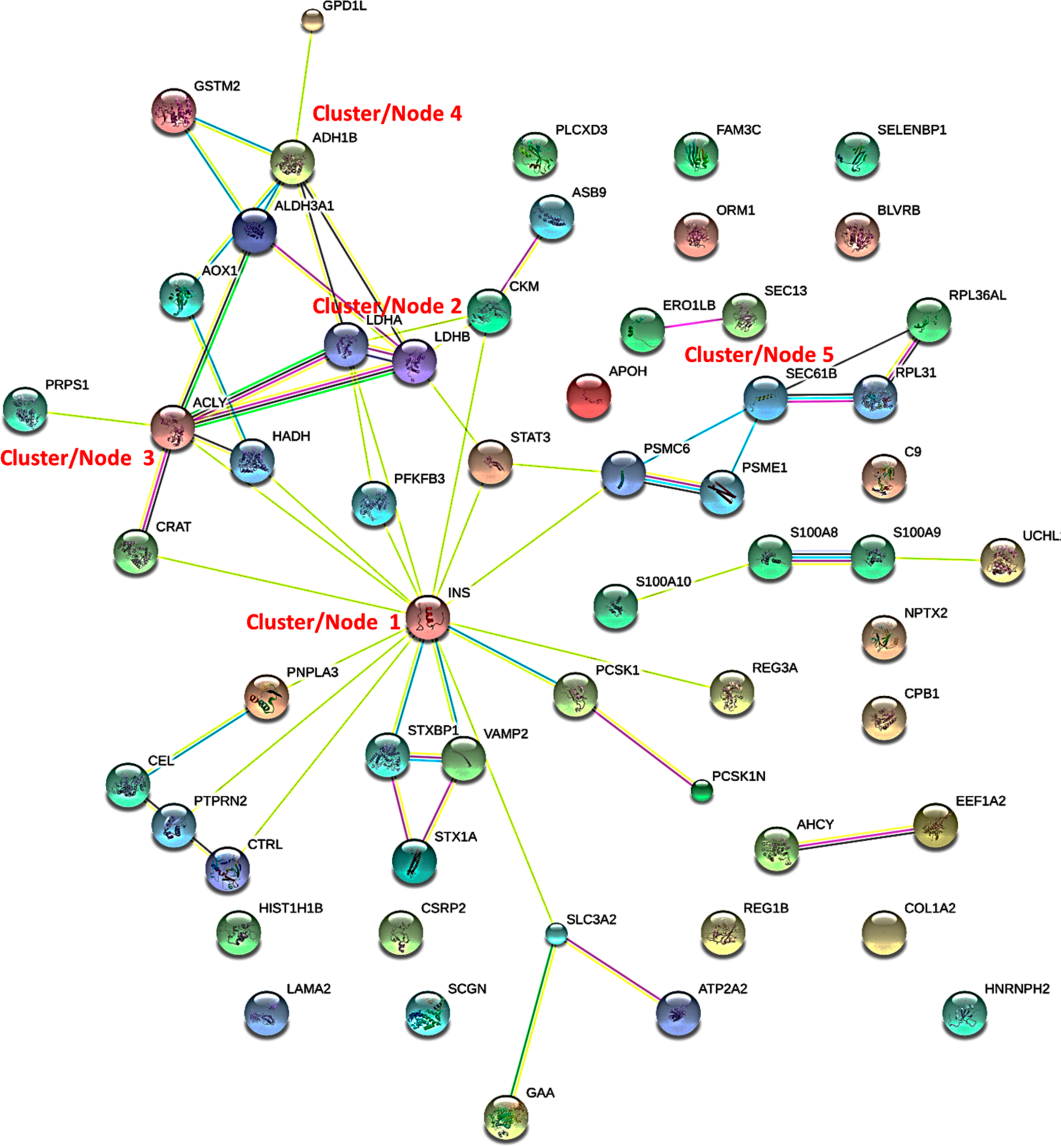
STRING analysis (http://www.string-db.org) derived protein-protein interaction networks for 63 islet proteins that are differentially regulated in T1D compared to ND cases. The network nodes represent proteins. Splice isoforms or post-translational modifications are collapsed, i.e., each node represents all proteins produced by a single, protein coding gene. Edges represent protein-protein associations. The associations are meant to be specific and meaningful with associated proteins jointly contributing to a shared function. This does not necessarily mean that the proteins are physically binding each other [18] (http://www.string-db.org).

**Fig 5:**
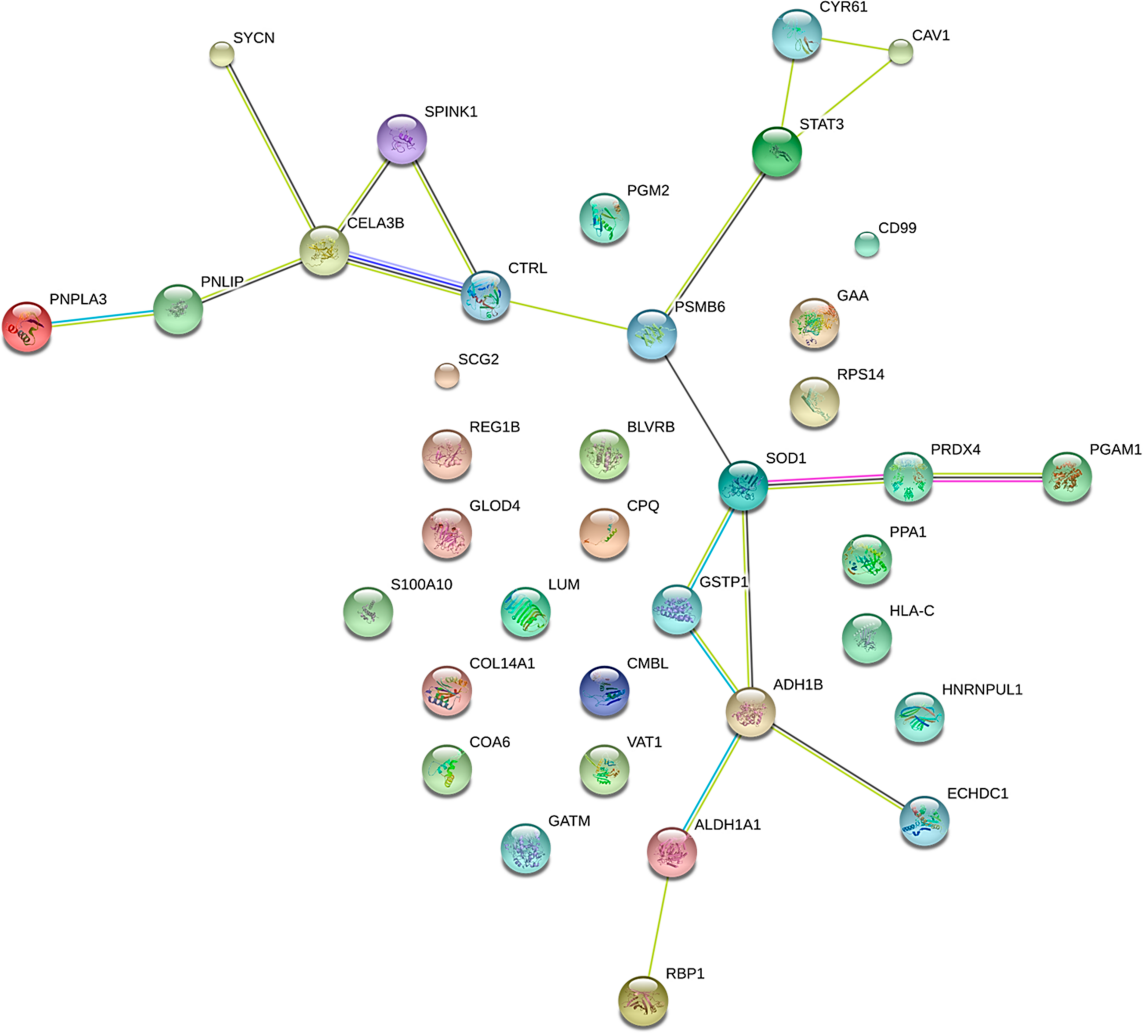
STRING analysis (http://www.string-db.org) derived protein-protein interaction networks for 39 islet proteins that are differentially regulated in AAB+ compared to ND cases. The network nodes represent proteins. Splice isoforms or post-translational modifications are collapsed, i.e., each node represents all proteins produced by a single, protein coding gene. Edges represent protein-protein associations. The associations are meant to be specific and meaningful with associated proteins jointly contributing to a shared function. This does not necessarily mean that the proteins are physically binding each other [18] (http://www.string-db.org).

We have performed a protein-protein interaction network analysis of the 63 differentially regulated proteins between T1D and non-diabetic cases. Fig 4 shows the interaction networks for differentially expressed islets as determined by STRING analysis [18]. The main cluster (1) or node for the interactions is insulin which is downregulated in T1D islets versus ND. The other node or cluster (2) is associated with lactate dehydrogenase A and B which are both upregulated in the T1D cases compared to ND. These are enjoined to a cluster (3) that has ATP-citrate synthase (ACYL) which is the primary enzyme responsible for the synthesis of cytosolic acetyl-CoA in many tissues. ACYL has a central role in de novo lipid synthesis. These interact with a cluster (4) that comprises alcohol dehydrogenases (ALDHs) proteins that play a major role in the detoxification of alcohol-derived acetaldehyde and are also involved in the metabolism of corticosteroids, biogenic amines, neurotransmitters, and lipid peroxidation. The final cluster 5 comprises of SEC61B which is necessary for protein translocation in the endoplasmic reticulum. There are also a number of proteins which do not form clusters. These include BLVRB, C9, COL1A2, CPB1, CRP2, FAM3C, HNRNPH2, LAMA2, NPTX2, ORM1, PLCXD3, SELENBP1, and SCGN. We have also performed STRING analysis on the proteins that are differentially expressed in AAB+ versus non-diabetic cases. The interaction network and clusters are different from the ones that are observed in T1D other than the cluster that includes alcohol dehydrogenases (Fig 5). The central cluster is focused on SOD1, which destroys radicals which are produced within the cells and are toxic to cells.

IPA network analysis was performed to identify potential markers that are differentially upregulated in only in AAB+ and T1D versus ND cases. The Top Canonical Pathways, Top Diseases and Bio Functions, Molecular and Cellular Functions, Physiological System Development and Function predicted for the proteins/genes that are differentially regulated in T1D and AAB+ and the probability values are summarized in Tables 1 and 2 respectively. The top 10 upregulated and downregulated genes that are included in the IPA analysis are shown in Tables 3 and 4, and the entire list is given in S2 and S3 Tables. Some of these uniquely upregulated molecules and activated pathways may serve as novel targets for prevention and treatment of T1D.

**Table 1:** Summary of IPA Analysis AAB+ versus non-diabetic cases.

**Top canonical pathways**		
Name	p-value	Overlap
Retinol biosynthesis	2.86E-05	8.3% 3/36
Glycogen degradation III	1.75E-04	16.7% 2/12
Retinoate biosynthesis I	1.21E-03	6.5% 2/31
Triacylglycerol degradation	1.29E-03	6.2% 2/32
Antigen presentation pathway	1.72E-03	5.4% 2/37
**Top upstream regulators**		
Upstream regulator	p-value of overlap	Predicted activation
NPC	1 8.22E-05	
SMARCA4	4.91E-04	Activated
HMGA1	7.30E-04	
PRKCE	9.09E-04	
TWIST1	1.18E-03	
**Top diseases and bio functions**		
**Diseases and disorders**		
Name	p-value	#Molecules
Endocrine system disorders	4.23E-02–2.01E-06	15
Gastrointestinal disease	4.34E-02–2.01E-06	14
Inflammatory disease	4.34E-02–2.01E-06	12
Inflammatory response	4.34E-02–2.01E-06	12
Organismal injury and abnormalities	4.96E-02–2.01E-06	26
**Molecular and cellular functions**		
Name	p-value	#Molecules
Cellular growth and proliferation	4.23E-02–2.09E-05	9
Cell death and survival	4.74E-02–1.22E-04	9
Cellular movement	4.07E-02–4.15E-04	5
Free radical scavenging	4.54E-02–4.99E-04	2
Small molecule biochemistry	4.82E-02–1.38E-04	10
**Physiological system development and function**		
Name	p-value	#Molecules
Behavior	3.32E-03–2.40E-04	2
Cell-mediated immune response	2.63E-02–1.66E-03	1
Connective tissue development and function	3.27E-02–1.66E-03	6
Embryonic development	4.07E-02–1.66E-03	4
Hematological system development and function	3.77E-02–1.66E-03	4

**Table 2:** Summary of IPA analysis T1D versus non-diabetic cases.

**Top canonical pathways**		
Name	p-value	Overlap
LXR/RXR activation	1.52E-05	4.1% 5/121
FXR/RXR activation	3.09E-04	3.2% 4/125
Role of IL-17A in psoriasis	5.06E-04	15.4% 2/13
Acute phase response signaling	9.40E-04	2.4% 4/168
Acetyl-CoA biosynthesis III (from citrate)	2.60E-03	100.0% 1/1
**Top upstream regulators**		
Upstream regulator	p-value of overlap	Predicted activation
PKM	1.16E-03	
VAV	2.41E-03	
ASB9	2.41E-03	
ATP2B4	2.41E-03	
MTOR	3.03E-03	
**Top diseases and bio functions**		
**Diseases and disorders**		
Name	p-value	#Molecules
Hematological disease	1.35E-02–1.37E-06	8
Metabolic disease	4.57E-02–1.37E-06	16
Endocrine system disorders	4.57E-02–1.82E-05	9
Cancer	4.61E-02–3.95E-05	8
Organismal injury and abnormalities	4.93E-02–3.95E-05	30
**Molecular and cellular functions**		
Name	p-value	#Molecules
Carbohydrate metabolism	4.07E-02–2.49E-06	10
Molecular transport	2.43E-02–2.49E-06	13
Small molecule biochemistry	4.32E-02–2.49E-06	15
Lipid metabolism	4.32E-02–6.57E-05	8
Cell-to-cell signaling and interaction	4.82E-02–1.38E-04	11
**Physiological system development and function**		
Name	p-value	#Molecules
Humoral immune response	9.84E-05–9.84E-05	2
Hematological system development and function	4.82E-02–1.55E-04	7
Immune cell trafficking	4.82E-02–1.55E-04	6
Connective tissue development and function	2.82E-02–1.22E-03	5
Cell-mediated immune response	4.07E-02–2.60E-03	1

**Table 3:** Ten top differentially regulated molecules in IPA analysis T1D vs ND.

Molecules	Expression Value	Molecules	Expression Value
S100A9	**↑**2.9	PFKB2	**↓**-2.9
REG1B	**↑**2.8	HADH	**↓**-2.3
REG3A	**↑**2.3	ATP2A3	**↓**-2.0
S100A8	**↑**2.3	STX1A	**↓**-1.9
C9	**↑**1.8	INS	**↓**-1.8
ABHD14B	**↑**1.7	SEC61B	**↓**-1.6
ALDH1B1	**↑**1.7	ASB9	**↓**-1.5
CKB	**↑**1.6	PLCXD3	**↓**-1.5
HIST1H1B	**↑**1.6	UCHL1	**↓**-1.5
ORM1	**↑**1.5	GSTM2	**↓**-1.5

**Table 4:** Ten top differentially regulated molecules in IPA analysis AAB+ vs ND.

Molecules	Expression Value	Molecules	Expression Value
REG1B	**↑**3.3	SYCN	**↓**-2.7
SPINK1	**↑**2.9	CTRL	**↓**-2.6
PSMB6	**↑**2.1	BLVRB	**↓**-2.1
SOD1	**↑**2.1	CAV1	**↓**-1.6
GSTP1	**↑**1.8	COA3	**↓**-1.4
CD99	**↑**1.8	HNRNPUL1	**↓**-1.3
GAA	**↑**1.6	PNLIPRP1	**↓**-1.2
CMBL	**↑**1.6	STAT3	**↓**-1.2
PGM1	**↑**1.6	CELA3B	**↓**-1.2
CUZD1	**↑**1.6	ECHCD3	**↓**-1.0

The most significantly upregulated protein in T1D cases with a 2.9 fold increase when compared to controls was S100A9. This gene is a member of the S100 family of proteins containing 2 EF-hand calcium-binding motifs. S100A9 is involved in a wide variety of intracellular and extracellular functions [19]. The protein plays a prominent role in the regulation of inflammatory processes, immune response and antimicrobial response [20–23]. It can induce neutrophil chemotaxis, adhesion, and increase the bactericidal activity of neutrophils. S100A8/A9 protein complex also binds arachidonic acid was recently identified by in vitro studies as a novel partner of the phagocyte NADPH oxidase [24]. S100A9 has a secretory sequence and its extracellular functions involve proinfammatory, antimicrobial, oxidant-scavenging and apoptosis-inducing activities. Its proinflammatory activity includes recruitment of leukocytes, promotion of cytokine and chemokine production, and regulation of leukocyte adhesion and migration [25–35]. S100A9 is proposed to also direct selective inflammatory stimulus-dependent S-nitrosylation of multiple targets [36]. It is important to note that S100A8 which is also a member of the S100 family is also significantly upregulated with a 2.2 fold increase in T1D cases versus the non-disease controls. Our current data is supported by a recent report that demonstrates the elevation of both S100A9 and S100A8 in the salivary proteome and peptidome profile in selected subjects with type 1 diabetes [37]. It is important to note that both S100A8/S100A9 are potential biomarkers and targets for therapeutics against autoimmune diseases [19, 38–40]. S100A8/S100A9 have also been associated with host defence mediation in neuropathic foot ulcers in patients with type 2 diabetes mellitus [41]. We have performed immonohistochemical validation for the expression of S100A9 in pancreas tissue sections from non-diabetic and T1D cases. Whole tissue lysates of non-diabetic and T1D show no difference in their expression of S100A9, however, there is a significant upregulation of S100A9 in the islets of T1D donors upon isolation of proteins using LCM (S1 Fig).

The other significantly upregulated proteins include Regenerating Islet-Derived 1 Beta (REG1B) and Regenerating Islet-Derived Protein III-Alpha (REG3A). The Reg gene family is a multigene family grouped into four subclasses, types I, II, III and IV based on the primary structures of the encoded proteins. Reg1B is associated pancreas regeneration is upregulated in human β cells under inflammatory conditions through the JAK/STAT pathway and upon upregulation may function as a growth factor for β cells to facilitate proliferation [42]. In addition REG3A is overexpressed during the acute phase of pancreatitis and in some patients with chronic pancreatitis [43]. We have previously reported the upregulation of REG3A in whole pancreata tissue lysates from nPOD samples from people with T1D [9]. The deterioration of β cells in the pancreas is a crucial factor in the progression of type 1 diabetes and therefore the recovery of β cells is critical for effective diabetic therapeutic strategies. The significant upregulation of REG1B and REG3A in the T1D cases would imply that these proteins may play a role in the persistence of beta cell mass in some people with T1D. Our current data is supports the subtractive hybridization studies by studies by Cho et al. that propose that REG3A is a potential target for genetic therapy in diabetes [44]. REG3A is also markedly increased in rats during pregnancy and is involved in islet regeneration and neogenesis [45–46].

The other significantly upregulated protein was complement 9 (C9), a sub-unit of the membrane attack complex (MAC) that is involved in innate and adaptive immune response mechanisms that destroy target cells by forming pores on their plasma membrane. C9 is 1.8 fold upregulated in T1D islets compared to the ND cases. Our observation is significant given that C9 and complement activation has been demonstrated to play a role in the lysis and destruction of transplanted pancreatic islets [47].

The other significantly upregulated protein includes ALDH1B1 which is a member of the alcohol dehydrogenase protein family that plays a role in the detoxification of alcohol-derived acetaldehydes. ALDH1B1 family of proteins are also involved in the metabolism of corticosteroids, biogenic amines, neurotransmitters, and lipid peroxidation. ALDH1B1 is 1.7 fold up regulated in T1D cases compared to the non-diabetic controls. The significance of this observation needs further studies although it has been demonstrated that there is a potential link between ALDH1B1and diabetes [48]. It is possible that its role in metabolism of lipid peroxidation is relevant given the role of 12-lipoxygenase in T1D [49]. The other significantly top upregulated proteins include abhydrolase domain containing 14B (ABHD14B)], creatine kinase B-type (CKB), HIST1H1B (Histone H1.5) and ORM1 (also known as alpha-2-glycoprotein) are significantly upregulated in T1D cases compared to the controls. ORM1 is involved in modulating the activity of the immune system during the acute-phase reaction with the degranulation of neutrophils.

In addition to insulin some of the other most significantly downregulated protein between T1D and the control cases is PFKB2 which is involved in glycolysis, hydroxyacyl-CoA dehydrogenase (HADH) which is involved in the beta-oxidation pathway and ATP2A3, magnesium-dependent enzyme catalyzes the hydrolysis of ATP coupled with the transport of calcium and STX1A, syntaxin 1A which is involved in peptide hormone metabolism and insulin secretion [50]. The others are SEC61B which is involved in the translocations of proteins to the ER, ASB9 which is involved in the ubiquitination pathway, PLCXD3 which has signal transducer and phosphoric diester hydrolase activity, UCHL1 which is a ubiquitin-protein hydrolase involved both in the processing of ubiquitin precursors and of ubiquitinated proteins and GSTM2 which is involved in the detoxification of electrophilic compounds and products of oxidative stress, by conjugation with glutathione.

In the AAB+ cases the most significantly upregulated protein is REG1B which is also upregulated in T1D cases. In addition the other uniquely upregulated proteins include SPINK1, a serine protease inhibitor which exhibits anti-trypsin activity, PSMB6 that is involved in ATP-dependent proteolytic activity, SOD1 which is involved in the detoxification of free radicals and GSTP1 which performs similar functions. CD99 is involved in leukocyte migration and T-cell adhesion and the protein is a beta cell surface marker [51]. The other significantly upregulated protein is GAA (Lysosomal alpha-glucosidase) which is essential for the degradation of glycogen to glucose in lysosomes; CMBL which is a cysteine hydrolase, CUZD1 and PGM1 which participates in glucose synthesis and catabolism. Polymorphisms in PGM1 have been associated with gestational diabetes in expectant mothers and juvenile onset of diabetes [52–55]. The most significantly downregulated proteins in the AAB+ cases include SYCN, CTRL, BLVRB, CAV1, COA3, HNRNPUL1, PNLIPRP1, STAT3, CELA3B, and ECHCD3. SYCN localizes to zymogen granules and is associated with insulin secretion.

Two previous studies have utilized LCM and mass spectrometry based proteomics to analyze differential protein expression in human islets obtained under different conditions and pathological states [56–57]. Unlike our current study, Nishida et al. [56], performed qualitative proteomic analysis to evaluate protein expression using the islets isolated from formalin-fixed paraffin-embedded pancreatic tissues from three autopsied cases of enterovirus-associated fulminant type 1 diabetes (FT1DM) with extensive insulitis compared to five autopsied non-diabetic men. On the other hand, Zhang et al. [57], recently performed comparative quantitative proteomic analyses of enzyme isolated cultured islets, and laser capture microdissected human islets and acinar tissue from fresh-frozen pancreas sections from three non-diabetic cadaveric donors. Unlike these two previous reports, our current proteomics data is obtained from 3 groups of disease stratified phenotype matched comprehensively annotated pancreas tissue samples from nPOD. Nishida et al, identified at total of 300 different proteins whereas Zhang et al identified at total of 1104 unique proteins. Therefore our current study which identifies a total of 2032 different unique protein groups, out of which 1491 proteins were quantified between ABB+ and T1D cases versus the cases with no evidence for disease, provides a very comprehensive proteome obtained from islets obtained after LCM. We have performed a comparative qualitative analysis between the data generated by Nishida et al, which analyzes disease stratified samples, and is most closely related to our current study. We have combined the list of proteins that Nishida, et al, identified in both islets affected by fulminant type 1 diabetes and in non-diabetic control pancreatic islets with those that they identified only in non-diabetic control pancreatic islets, and compared these proteins to the 2032 unique proteins that are identified in this study. S2 Fig summarizes the overlap between the two studies. There is a very highly significant overlap between the proteins that were identified by Nishida et al compared to our current study. 9 proteins KRT10, KRT2, RCL, YCN, SRSF8, ALB, PKM2, KRT9, HISTH2AB and KRT19 are exclusively identified in the previous study. It is important to note that a majority of these proteins including KRT10, KRT2, ALB, KRT9 and KRT19 are often considered as contaminants in proteomics experiments and are always excluded in comparative analyses. Taken together our data provides a very comprehensive proteomic analysis of disease stratified LCM islets to date.

## Conclusion

In this study, we report the successful analysis of protein expression in islets acquired from well annotated nPOD pancreas tissue samples by laser capture micro-dissection and in-depth proteomic analysis using mass spectrometry. The study provides comprehensive qualitative and quantitative information on the proteins expressed in islets and during T1D progression. This may provide pathomechanistic insights and provide the rationale and perspectives for novel therapeutic targets for intervention in T1D. The next steps would be to evaluate the presence and levels of the lead protein candidates in well characterized patient cohort samples from ongoing or completed studies evaluating the natural history and triggers of T1D.

## Supporting information

S1 Fig S100A9 localizes to islets in T1D donor pancreas tissues, but in the exocrine tissue of non-diabetic donors. Representative images from nPOD donors 6073 (ND), 6076 (T1D) and 6077 (T1D) are shown. Whole tissue lysates of non-diabetic and T1D show no difference in their expression of S100A9, however, there is a significant upregulation of S100A9 in the islets of T1D donors upon isolation of proteins using Laser Capture Microdissection. Glucagon (green) and S100A9 (red) immunofluorescent staining were carried out as described in the materials and methods section.
(TIF)

S2 Fig Qualitative comparative analysis of 2032 unique proteins that are identified in this study (Nyalwidhe et al. 2017) and the proteins that are identified by Nishida et al. 2014. The proteins by identified by Nishida et al. include those identified in islets affected by fulminant type 1 diabetes and in non-diabetic control pancreatic islets those that are identified only in non-diabetic control pancreatic islets. A highly significant overlap exists between the proteins that are identified in the two studies.
(TIF)

S1 Table Donor phenotype and pancreas tissue samples used in the study. (DOCX)

S2 Table Differentially expressed proteins in islets from non-diabetic and AAB+ cases. (XLSX)

S3 Table Differentially expressed proteins in islets from non-diabetic and Type 1 diabetes cases. (XLSX)

S4 Table Qualitative and quantitative comparisons between non-diabetes versus autoantibody cases and T1D cases. (XLSX)

S5 Table Functional annotation and enrichment of differentially expressed proteins between T1D versus non-diabetes cases. (XLSX)

S6 Table Functional annotation and enrichment of differentially expressed proteins between AAB+ versus non diabetes cases. (XLSX)
