# Supplementary figures and images for: Comparative quantitative proteomic analysis of disease stratified laser captured microdissected human islets identifies proteins and pathways potentially related to type 1 diabetes
Source: PLoS One. 2017 Sep 6;12(9):e0183908. doi: 10.1371/journal.pone.0183908 (PMC5587329; doi:10.1371/journal.pone.0183908)

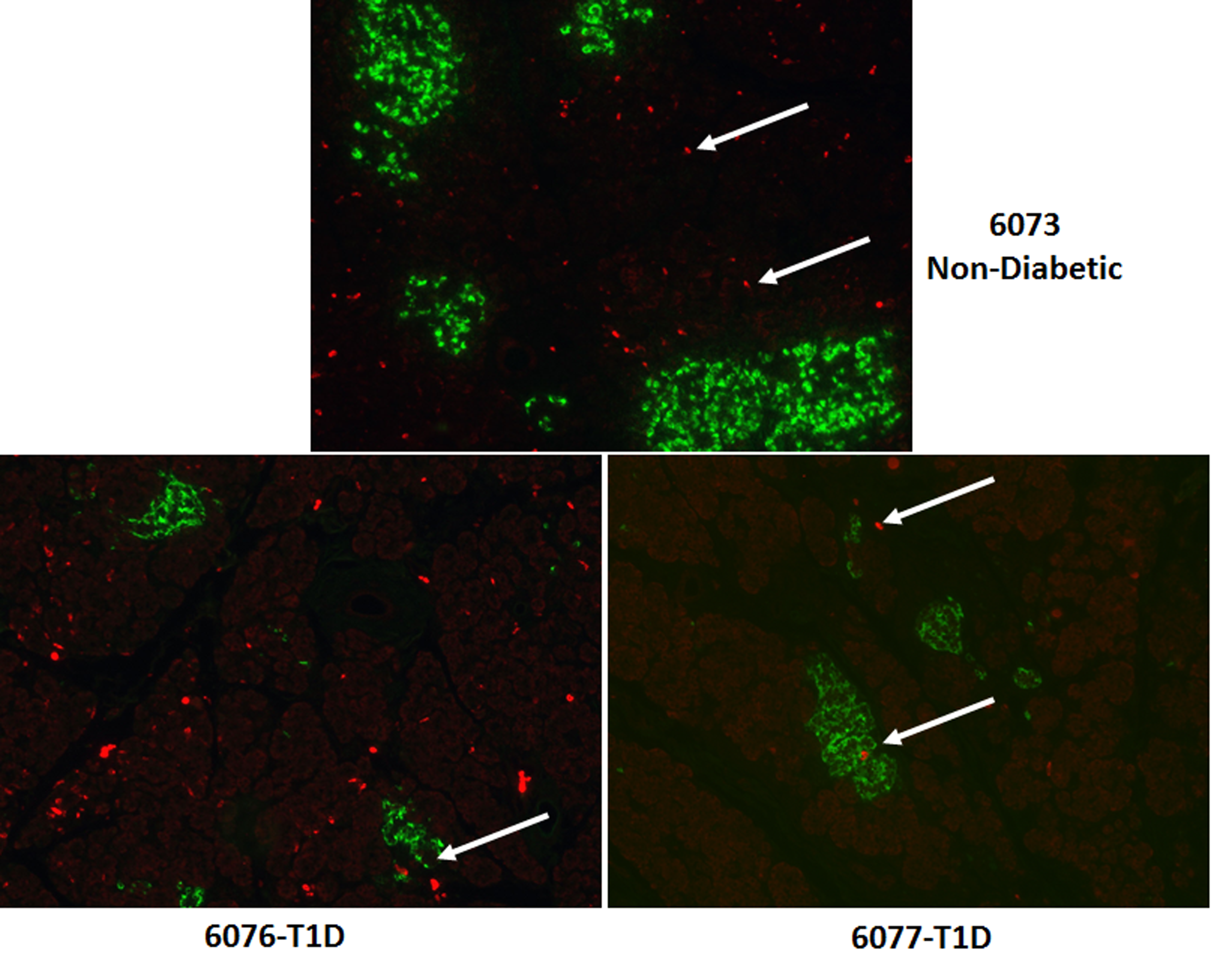

Supplement: S1 Fig — Representative images from nPOD donors 6073 (ND), 6076 (T1D) and 6077 (T1D) are shown. Whole tissue lysates of non-diabetic and T1D show no difference in their expression of S100A9, however, there is a significant upregulation of S100A9 in the islets of T1D donors upon isolation of proteins using Laser Capture Microdissection. Glucagon (green) and S100A9 (red) immunofluorescent staining were carried out as described in the materials and methods section. (TIF) [file pone.0183908.s001.tif]

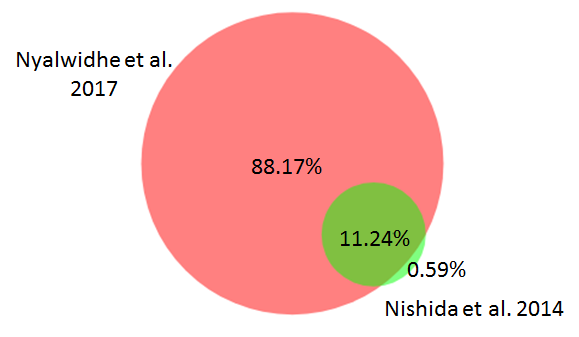

Supplement: S2 Fig — The proteins by identified by Nishida et al. include those identified in islets affected by fulminant type 1 diabetes and in non-diabetic control pancreatic islets those that are identified only in non-diabetic control pancreatic islets. A highly significant overlap exists between the proteins that are identified in the two studies. (TIF) [file pone.0183908.s002.tif]
