# Supplementary material for: Comparative quantitative proteomic analysis of disease stratified laser captured microdissected human islets identifies proteins and pathways potentially related to type 1 diabetes
Source: PLoS One. 2017 Sep 6;12(9):e0183908. doi: 10.1371/journal.pone.0183908 (PMC5587329; doi:10.1371/journal.pone.0183908)
Supplement: S1 Table — (DOCX) [file pone.0183908.s003.docx]

| **nPOD Case ID** | **Donor Type** | **AutoAb (RIA) Results** | **Age (Years)** | **Gender** | | **Diabetes Duration (years)** | **C-peptide (ng/ml)** | **BMI** |
| --- | --- | --- | --- | --- | --- | --- | --- | --- |
| 6234 | ND | Negative | 20 | | F | - | 6.89 | 25.6 |
| 6238 | ND | Negative | 20 | | M | - | 1.17 | 21.7 |
| 6289 | ND | Negative | 19 | | M | - | 8.05 | 38.3 |
| 6318 | ND | Negative | 10 | | F | - | 3.89 | 17.6 |
| 6339 | ND | Negative | 23.3 | | M | - | 10.56 | 25 |
| 6158 | AAB+ | mIAA, GADA | 40.3 | | M | - | 0.51 | 29.7 |
| 6167 | AAB+ | IA-2A, ZnT8A | 37 | | M | - | 5.43 | 26.3 |
| 6184 | AAB+ | GADA+ | 47.6 | | F | - | 3.42 | 27 |
| 6267 | AAB+ | GADA+,IA-2A+ | 23 | | F | - | 16.59 | 23.5 |
| 6175 | T1D | GADA+ | 43 | | M | 12 | 3.48 | 19.9 |
| 6245 | T1D | GADA+,IA-2A+ | 22 | | M | 7 | < 0.05 | 21.1 |
| 6247 | T1D | mIAA | 24 | | M | 0.6 | 0.47 | 24.3 |
| 6325 | T1D | GADA+,IA-2A+, mIAA+ | 20 | | F | 6 | 0.14 | 31.2 |

**S1 Table.** Donor phenotype and pancreas tissue samples used in the study.
